# Identification of stromal cell-derived factor 4 as a liquid biopsy-based diagnostic marker in solid cancers

**DOI:** 10.1038/s41598-023-42201-2

**Published:** 2023-09-20

**Authors:** Takahiro Shinozuka, Mitsuro Kanda, Dai Shimizu, Shinichi Umeda, Hideki Takami, Yoshikuni Inokawa, Norifumi Hattori, Masamichi Hayashi, Chie Tanaka, Goro Nakayama, Yasuhiro Kodera

**Affiliations:** Department of Gastroenterological Surgery, Nagoya University Graduate School of Medicine, 65 Tsurumai-Cho, Showa-Ku, Nagoya, 466-8550 Japan

**Keywords:** Cancer, Cancer screening, Gastrointestinal cancer

## Abstract

There is a need for serum diagnostic biomarkers to improve the prognosis of solid malignant tumors. Here, we conducted a single-institutional study to evaluate the diagnostic performance of serum stromal cell-derived factor 4 (SDF4) levels in cancer patients. Serum samples were collected from a total of 582 patients with solid cancers including gastric cancer (GC) and 80 healthy volunteers. SDF4 protein levels in sera, and conditioned media or lysates of human GC cell lines were measured by enzyme-linked immunosorbent assay, and those in GC tissue by immunohistochemistry. Serum SDF4 levels were higher in patients with cancer than the healthy control in all cancer type. Regarding GC, serum SDF4 levels distinguished healthy controls from GC patients with the area under the curve value of 0.973, sensitivity of 89%, and specificity of 99%. Serum SDF4 levels were significantly elevated in patient with early stage GC. In immunohistochemistry, the frequency of SDF4-positive GC tumors did not vary significantly between GC stages. The ability of human GC cell lines to both produce and secrete SDF4 was confirmed in vitro. In conclusion, serum SDF4 levels could be a promising candidate for a novel diagnostic biomarker for GC and other malignancies.

## Introduction

Early detection of cancer is crucial to reducing cancer mortality worldwide. In recent years, various liquid-based materials, including exosomes, circulating tumor DNA, and serum noncoding RNAs, have been developed as diagnostic biomarkers; however, the measurement is generally technically complex and expensive, which hinders their utility in clinical applications^1^. Thus, there is still a need for identifying accurate serum diagnostic biomarkers that can be routinely and inexpensively measured for large-scale screening of early stage cancer, particularly solid cancers^2^.

Gastrointestinal cancers such as gastric cancer (GC) and colorectal cancer have high mortality rates worldwide^3^. Endoscopy has been the standard method for screening and diagnosis of gastrointestinal cancers, but it has several drawbacks, such as its invasive nature, risk of complications, time-intensive tissue evaluation, and excessive cost. Serological tumor biomarkers are desirable for cancer screening in terms of the easy and relatively non-invasive method of sample collection and low cost^4^. However, conventional serum tumor markers such as carcinoembryonic antigen (CEA) and carbohydrate antigen 19-9 (CA19-9) are not satisfactory due to their relatively low sensitivity and specificity^5,6^. In the case of GC, there is a particularly clear need for a serum diagnostic marker that is elevated in patients with early stage GC compared with healthy individuals.

We employed a dataset of previously reported secretome analyses using GC cell lines to identify new diagnostic biomarkers that could be secreted to circulation^7^ and further screened candidate proteins using two public databases, the human cancer secretome database (HCSD) and The Cancer Genome Atlas (TCGA) database^8,9^. A pilot experiments were performed to measure blood levels of candidate proteins in healthy controls and GC patients, and we found that stromal cell-derived factor 4 (SDF4) had the best diagnostic ability for GC. Therefore, SDF4 was selected as the final candidate for a diagnostic biomarker in this study, as a potential serum diagnostic tumor marker for GC and other cancers (breast cancer, colorectal cancer, pancreatic cancer, esophageal cancer and hepatic cancer).

SDF4 is a 45 kDa Ca^2+^-binding protein expressed by various normal human tissues, including gastric tissues^10,11^. Some studies have reported that SDF4 is associated with Ca^2+^-dependent secretory pathways and may be related to the progression of certain cancers; however, to the best of our knowledge, there have been no previous studies of serum SDF4 levels and their clinicopathological relevance in cancer patients^12–14^.

The aim of this study was to evaluate the diagnostic significance of serum SDF4 levels in cancer patients, with a particular focus on GC. We also investigated the secretion of SDF4 in human GC cell lines, the distribution of SDF4 in primary GC tissues.

## Results

### Identification of SDF4 as a candidate for a diagnostic biomarker

We employed a dataset of previously reported secretome analyses using GC cell lines to identify new diagnostic biomarkers that could be secreted to circulation^7^. This dataset yielded 188 candidate-secreted proteins. Of them, 152 proteins were confirmed to be expressed in the body fluids according to the HCSD^8^. We further investigated whether their expression levels are increased in the GC tissues compared to normal gastric mucosa using TCGA data and selected 79 candidate proteins^9^. Among these proteins, ten proteins had not been investigated in the field of cancer biomarkers by a literature review. A pilot experiments were performed to measure blood levels of 10 candidate proteins in healthy controls (*N* = 18) and GC patients (*N* = 70), and we found that SDF4 had the highest the area under the curve (AUC) value in the receiver operating characteristic (ROC) curve analysis discriminating between healthy controls and GC patients (Supplemental Table 1). Therefore, SDF4 was selected as the final candidate for a diagnostic biomarker in this study.

### Patient characteristics

Serum samples were collected from a total of 582 patients with GC (*N* = 396), breast cancer (*N* = 30), colorectal cancer (*N* = 30), pancreatic cancer (*N* = 27), esophageal cancer (*N* = 75), and hepatic cancer (*N* = 24). The GC patient population consisted of 275 men and 121 women (Table 1) who had a median age of 67.5 years (range, 28–92 years) and a mean age of 66.9 years (standard deviation, 12.5). Histological subtypes of GC were divided into differentiated (*N* = 175; papillary, well differentiated, and moderately differentiated adenocarcinoma) and undifferentiated (*N* = 221; poorly differentiated adenocarcinoma, signet cell carcinoma, and mucinous carcinoma). According to the 8th edition of the Union for International Cancer Control (UICC) classification, 184, 57, 68, and 87 GC patients were at clinical stages I, II, III and IV, respectively.

**Table 1:** Characteristics of 396 gastric cancer patients.

Variables	Values
Age (years), mean ± standard deviation	66.9 ± 12.5
Sex (male/female)	275/121
Tumor location	
Upper third	108 (27%)
Middle third	166 (42%)
Lower third	121 (31%)
Tumor size (mm), mean ± standard deviation	41.8 ± 28.1
Differentiation	
Differentiated	175 (44%)
Undifferentiated	221 (56%)
Clinical T factor	
T1	148 (37%)
T2	102 (26%)
T3	101 (26%)
T4	45 (11%)
Clinical N factor	
N0	234 (59%)
N1	93 (23%)
N2	51 (13%)
N3	18 (5%)
Clinical stage	
I	184 (47%)
II	57 (14%)
III	68 (17%)
IV	87 (22%)

### Diagnostic value of serum SDF4 levels

**Figure 1:**
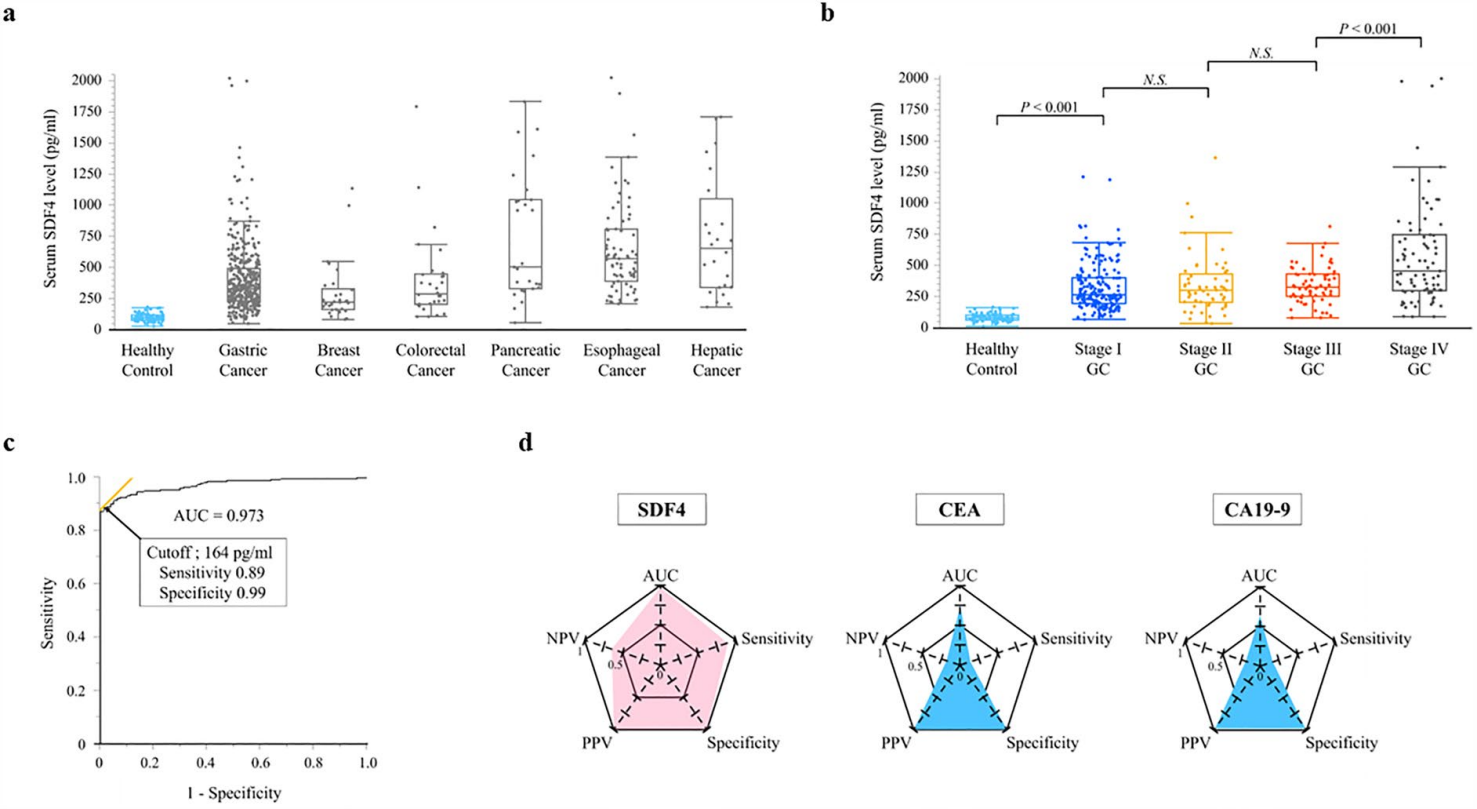
(**a**) Serum levels of SDF4 in healthy controls and patients with various cancers. (**b**) Serum levels of SDF4 in healthy controls and patients with stage I, II, III, and IV GC. (**c**) Receiver operating characteristic curve analysis to determine the serum SDF4 level able to discriminate between healthy controls and patients with GC. (**d**) Diagnostic performances of SDF4, CEA, and CA19-9 for identifying patients with GC.

The median serum SDF4 level for the 80 healthy controls was 83.8 pg/ml (range, 17.9–169.9 pg/ml) and higher values were obtained for all cancer patient groups (Fig. 1a). The median serum SDF4 levels in GC patients increased with clinical stage, and were 266.2 pg/ml (range, 70.4–1202 pg/ml), 303.4 pg/ml (range, 39.1–1353.1 pg/ml), 326.6 pg/ml (range, 84.1–808.8 pg/ml), and 455.1 pg/ml (range, 92.8–3185.3) in patients at clinical GC stages I, II, III, and IV, respectively (Fig. 1b). SDF4 levels were significantly higher in patients with stage I GC compared with healthy controls (*P* < 0.001) and in patients with stage IV GC compared with stage III GC (*P* < 0.001). ROC curve analysis identified an optimal cutoff SDF4 concentration of 164 pg/ml for distinguishing between healthy controls and GC patients, with a sensitivity of 89% and a specificity of 99% (Fig. 1c). Notably, the AUC value for SDF4 was 0.973, which was superior to the values for CEA (0.75) and CA19-9 (0.639). Figure 1d shows a summary of the diagnostic performances of SDF4, CEA, and CA19-9 in terms of AUC, sensitivity, specificity, positive predictive value (PPV), and negative predictive value (NPV). These data showed that serum SDF4 concentration had better diagnostic capability than either serum CEA or CA19-9 concentrations for GC patients. We examined the diagnostic ability of serum SDF4 levels for GC stratified by sex (male and female). The results of male and female subgroups were similar with those of the overall cohort (Supplemental Fig. 1).

### Prognostic value of serum SDF4 levels of GC patients

**Figure 2:**
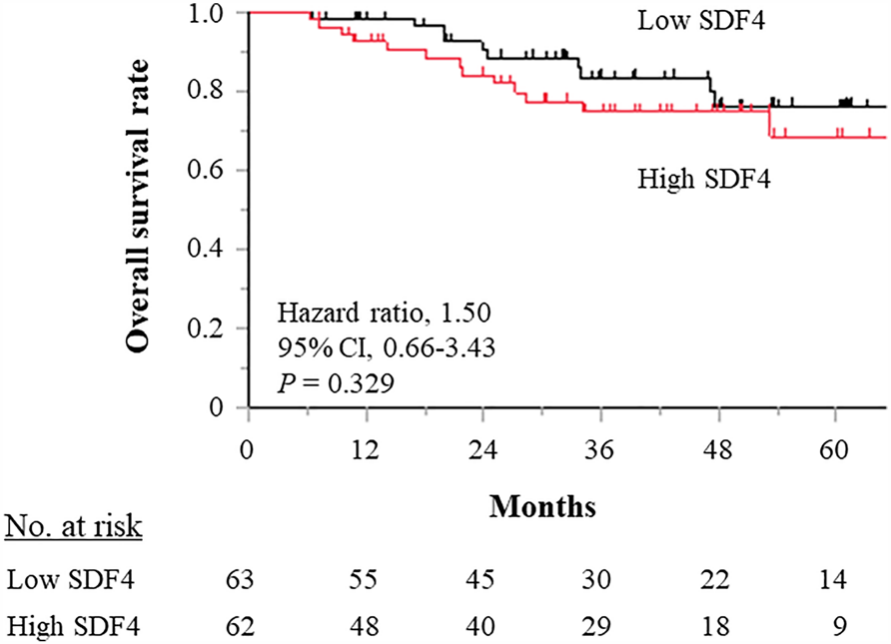
Kaplan–Meier analysis of overall survival in GC patients with high or low serum SDF4 levels based on the median serum level. The number at risk every 12 months is shown at the bottom of the figure.

To determine whether serum SDF4 levels might also have utility as a prognostic marker for GC, we compared the overall survival (OS) of patients with advanced GC in our cohort. Patients with stage II/III GC (*N* = 125) were assigned to two groups according to the median serum SDF4 value of 313.7 pg/ml (low SDF4 group, *N* = 63; high SDF4 group, *N* = 62). There was no significant difference in OS between the two groups (hazard ratio 1.50, 95% confidence interval 0.66–3.43; *P* = 0.329) (Fig. 2). The rate of adjuvant chemotherapy in the high SDF4 group was 54.84% (34/62 cases), and that of low SDF4 group was 57.14% (36/63 cases), respectively. There were no significant differences between the two groups (*P* = 0.795).

### SDF4 levels in GC cells lysates and conditioned medium

**Figure 3:**
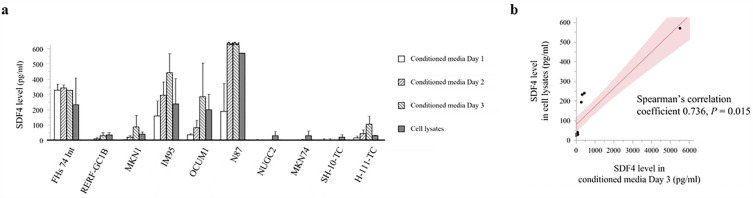
(**a**) Production of SDF4 by human GC and normal gastric cell lines measured by ELISA. SDF4 levels in conditioned media were quantified on days 1–3 of culture and cell lysates were prepared and quantified on day 3 of culture. (**b**) Correlation between SDF4 levels in conditioned medium and cell lysates of all cell lines on day 3.

To determine whether GC cells could be the source of circulating SDF4, we measured SDF4 levels in cell lysates and conditioned media from nine GC cell lines and a normal gastric epithelial cell line, FHs 74 Int, by enzyme-linked immunosorbent assay (ELISA) (Fig. 3a). Several of the GC cell lines secreted SDF4 over 72 h in culture, and a significant correlation was detected between intracellular and secreted levels of all cell lines measured on day 3 (Fig. 3a,b; Spearman’s correlation coefficient 0.736, *P* = 0.015).

### Immunohistochemistry staining of SDF4 in GC tissues

**Figure 4:**
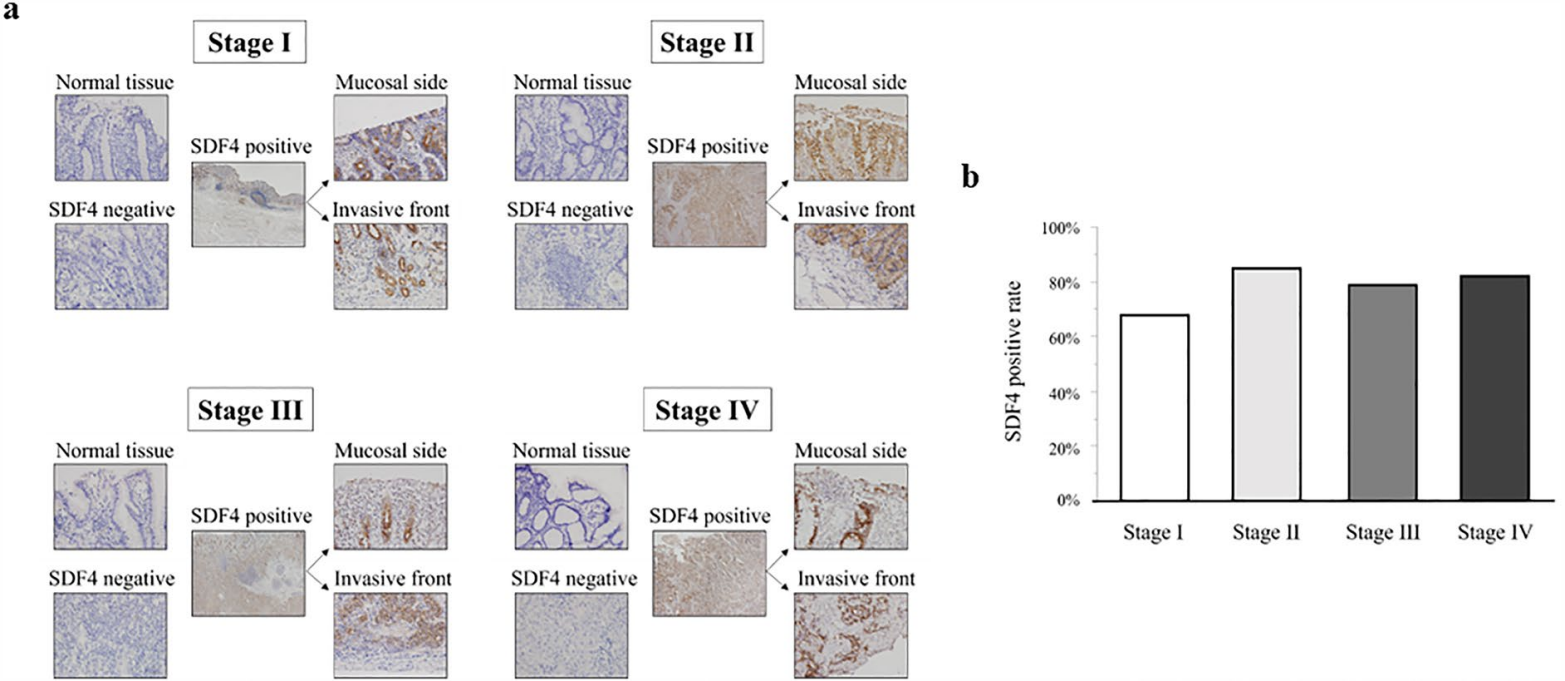
(**a**) Representative immunohistochemical staining of SDF4 in normal gastric and GC tissues. The five panels at each stage show normal gastric tissue, negative control GC tissue, positively stained GC tissue, and enlargements showing the mucosal side and invasive front. (**b**) Proportion of tumors at each stage staining positively for SDF4.

To confirm that GC tissue expresses SDF4 protein and to determine whether the expression pattern changes at various stages of disease, we performed immunohistochemistry of representative stage I, II, III, IV GC tissues as well as adjacent normal tissues (Fig. 4a). SDF4 was absent from the normal tissues (the positive staining rate was 0%), but in GC tissue sections, expression was observed in the cytoplasm of cancer cells but not in the tumor stroma. In SDF4-positive tumor tissues, both the mucosal side and the invasive front showed positive staining. The majority of tissue samples at all stages were positive for SDF4, and the positive staining rates were not significantly different between stages (stage I 66.7%, *N* = 18; stage II 84.6%, *N* = 13; stage III 78.6%, *N* = 14; stage IV 81.8%, *N* = 11) (Fig. 4b).

### Perioperative changes in serum SDF4 levels

**Figure 5:**
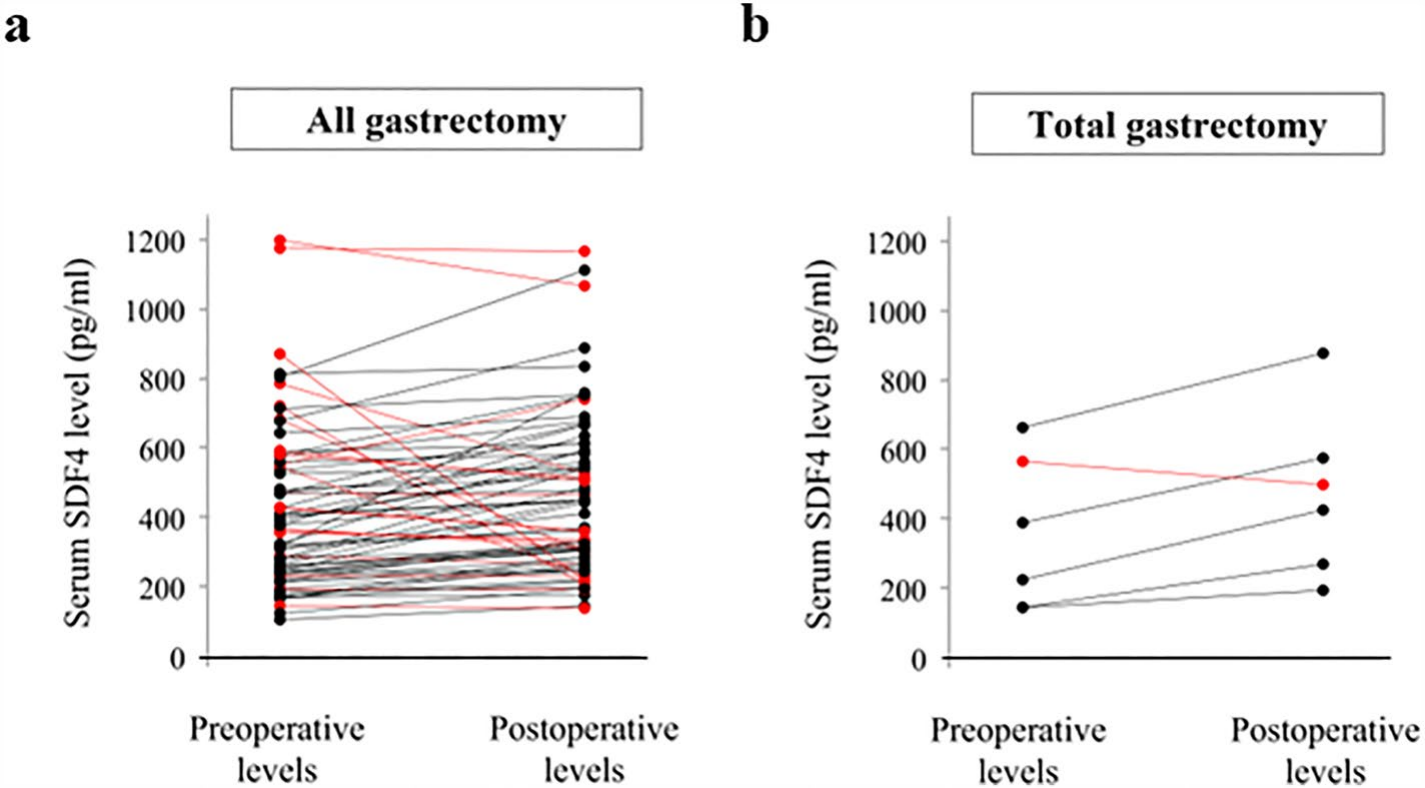
Perioperative changes in serum SDF4 levels in (**a**) patients undergoing any type of gastrectomy or (**b**) total gastrectomy.

We next determined whether serum SDF4 levels changed perioperatively by comparing preoperative serum samples with samples obtained within 3 months after R0 gastrectomy in 73 patients without GC recurrence (the acute inflammatory state is considered to be resolved within 3 months). Unexpectedly, we detected no significant change in serum SDF4 levels after resection of primary GC lesions (Fig. 5a) or even after total gastrectomy in the 6 patients who underwent total gastrectomy (Fig. 5b).

## Discussion

A diagnostic biomarker that requires an invasive procedure to obtain cancer tissue is unsuitable for large-scale screening^15^. In contrast, biomarkers evaluable using the blood samples, which can be collected minimally invasively, are more conducive to routine screening. Recently, several reports have identified circulating microRNAs or tumor DNA as promising biomarkers for early detection of cancer^16–21^. However, methods to evaluate these markers are complex and are liable to be too costly for routine clinical practice compared with quantification of a serum protein using a simple ELISA kit.

Stromal cell-derived factors (SDFs), a set of proteins originating from stromal cells, including fibroblasts, encompasses several members such as SDF1, SDF2, SDF3, SDF4, and SDF5^11,14^. SDF1 (also known as C-X-C motif chemokine 12) is well-characterized, while other SDFs are less thoroughly defined^10^. SDF4, also termed 45 kDa Ca^2+^-binding protein belongs to the CREC protein family, distinguished by the presence of six EF-hand motifs and calcium-binding motifs^22^. SDF4 is expressed by various normal human tissues, including gastric tissues, and localized in the cytosol, presented on the cell surface, or secreted into the extracellular space^13^. SDF4 is involved in the regulation of Ca^2+^-dependent secretory cargo sorting pathways within the trans-Golgi network^23^. An elevation in SDF4 expression has been reported in several cancer cell types, particularly those with enhanced proliferative and metastatic potential^13,24,25^.

We have previously reported on candidates of novel serum protein biomarkers for GC^26,27^, but either the sensitivity or the specificity were deemed unsatisfactory for further development. In the present study, we showed that serum SDF4 levels were elevated in patients with various solid cancer compared with the healthy control, and exhibited high sensitivity and specificity with a robust AUC value particularly in GC. We demonstrated that serum SDF4 levels were significantly elevated in patients with early stage GC compared with healthy subjects, as well as in patients with cancers representing different histological types than adenocarcinoma. These findings suggested that SDF4 may have utility as a serum biomarker for early screening of cancer, with patients exhibiting high serum SDF4 levels to be referred for additional examinations for GC and other cancers. Considering the potential of SDF4 as a diagnostic biomarker, which is applicable for various types of malignancies other than gastric cancer, would be beneficial in large-scale population screening programs.

In the present study, serum SDF4 levels were significantly higher in patients with stage I GC than in healthy controls. This result suggests that serum SDF4 levels do not correlate with tumor burden and may not be useful as a prognostic marker for patients with advanced GC. However, serum SDF4 levels were also significantly elevated in patients with stage IV GC *vs* stage III GC. Since stage IV GC includes a variety of tumor burden and clinical features such as peritoneal dissemination, gastrointestinal stenosis, and multiple liver metastases, making it difficult to fully explain the reason for high SDF4 values in stage IV GC. In fact, a large dispersion of serum SDF4 values was observed among stage IV GC patients, indicating further validation with a larger sample size is needed.

It is speculated that elevated serum SDF4 levels may be derived from cancer cells, other cells activated under inflammatory conditions, including cells within the tumor microenvironment, or normal tissues such as the liver^28,29^. Our in vitro experiments showed that human GC cell lines not only express SDF4 protein but also secrete it into the extracellular medium. Because the levels of secreted SDF4 correlated positively with the intracellular SDF4 levels, these findings suggest that the elevated serum levels of SDF4 may be derived from active secretion by live GC cells or by passive release from apoptotic GC cells. In addition, even if some GC cell lines produce little SDF4 in vitro, serum SDF4 levels could increase in vivo due to the heterogeneity of gastric cancer tissues or secretion from other organs. We note that secretion of SDF4 by the non-cancerous gastric epithelial cell line FHs 74 Int is consistent with the presence of SDF4 in the blood of healthy individuals. However, it is speculated that cells in the tumor microenvironment and other inflammatory tissues might promote the production and secretion of SDF4 by tumor cells, leading to abnormally elevated serum SDF4 levels at an early stage in GC development^26,27^.

Our immunohistochemical staining experiments showed that SDF4 protein is expressed in the cytoplasm of GC cells but not in the stroma or adjacent normal tissues, suggesting that serum SDF4 may indeed be derived from the GC tumor cells in vivo. However, we did not detect SDF4 in some of the GC tissue samples from patients who had elevated serum SDF4 levels. There are several possible explanations for this discrepancy. First, specimens were taken from one region of the tumor and they may not be representative of the entire tumor. Second, SDF4 may be largely produced and released into the circulation by non-cancerous tissues, such as remnant gastric mucosa or other normal tissue such as the liver, as discussed above. Our examination of the distribution of SDF4 in GC tissue revealed its presence at both the mucosal side and the invasive front at all GC stages. Thus, even as the tumor progressed, there was no shift in positive staining of SDF4 to one particular tumor region, which is consistent with the lack of correlation between elevated serum SDF4 levels and cancer stage.

We found that serum SDF4 levels measured before and 3 months after radical resection of GC were not significantly different. One possible explanation is that SDF4 may have a very long half-life and is not eliminated from the circulation for at least 3 months after the tumor has been excised. This would contrast with the situation for CEA, which has a mean half-life of approximately 10 days and would further suggest that serum SDF4 levels could not be used as a marker for GC recurrence after radical surgery^27,30^. Long-term follow-up and further basic research are required to investigate the changes in serum SDF4 levels and to understand physiological roles of SDF4 protein. A second explanation for the lack of change in serum SDF4 levels postoperatively is that it may be produced by remnant gastric tissues, including gastric intestinal dysplastic mucosa or gastritis mucosa^31,32^. However, this is an unlikely explanation because we found that serum SDF4 levels did not decrease postoperatively even after total gastrectomy. A third possibility is that the origin of serum SDF4 may switch to tissues other than gastric tissues postoperatively. Postoperative inflammation and induction of inflammatory cytokines may lead to elevated production by normal tissues such as the liver. However, it is difficult to collect normal tissue samples from postoperative patients and this possibility remains to be examined. Aikou et al. have reported that trefoil factor family could be a serum diagnostic marker of GC. Similarly, it did not decrease after gastrectomy and they also called for additional experiments to clarify the origin of that serum marker after gastrectomy^28^.

There are several limitations to this study. The sample size was small and was derived from a single Japanese institution and may therefore not be representative of other patient populations. Therefore, further analysis of a larger multi-institutional cohort will be necessary to determine the diagnostic significance of serum SDF4 and to translate our findings to clinical practice. Moreover, we need to develop a multiplex ELISA kit that can simultaneously measure SDF4 and other serum tumor markers, thereby increasing the sensitivity and specificity for early cancer diagnosis. External validation of the reproducibility of the kit and standardization across laboratories will also be essential. Finally, our results demonstrated the potential diagnostic utility of serum SDF4 levels but did not identify the cell/tissue origin or biological functions of SDF4 in cancer patients.

In conclusion, serum SDF4 levels may be a novel diagnostic biomarker for not only advanced but also early gastric cancer. We are planning further validation in larger cohorts that include patients of other races and ethnicities to validate its diagnostic performance and to develop ELISA kits that could be suitable to the large-scale screening of patients with various malignancies, including GC.

## Methods

### Serum samples from cancer patients and healthy volunteers

Patients with solid cancers (gastric *N* = 396*,* breast *N* = 30, colorectal *N* = 30, pancreatic *N* = 27, esophageal *N* = 75, and hepatic *N* = 24) who were seen at the Nagoya University Hospital Department of Gastroenterological Surgery between 2014 and 2020 were included in the study. The eligibility criteria (as in our previous study^27^) included histologically confirmed primary lesions and age ≥ 20 years. Exclusion criteria included a history of other malignancies or any condition that might render the patient unsuitable for the study at the discretion of the investigators. GC stage was classified according to the 8th edition of UICC^33^. Healthy adults (*N* = 80) who underwent annual medical examinations (physical examination, blood tests and chest X-rays) at Nagoya University Hospital and were not currently being treated for any condition were enrolled as the healthy control group. Serum samples (1 ml) were collected from volunteers or from cancer patients prior to treatment (within 28 days before surgery or induction of radiotherapy or systemic therapy and from patients who underwent R0 gastrectomy and had no recurrence of GC postoperatively (within 3 months after surgery). The blood samples are collected between 8:00 a.m. and noon at the outpatient clinic. Blood was collected into tubes containing serum-separating agents and maintained at 4 °C for at least 30 min. The tubes were then centrifuged at 3000 rpm for 5 min, and sera were transferred to fresh tubes and stored at − 80 °C until analyzed^34^. This study was conducted in accordance with the ethical guidelines of the World Medical Association Declaration of Helsinki–Ethical Principles for Medical Research Involving Human Subjects and was approved by the Ethics Committee of Nagoya University (approval number 2014-0043). Written informed consent was obtained from all participants.

### Cell lysates and conditioned media

Nine gastric cancer cell lines (RERF-GC1B, MKN1, IM95, OCUM1, N87, NUGC2, MKN74, SH-10-TC, and H-111-TC) and the non-tumorigenic gastric epithelial cell line FHs 74 Int were obtained from the American Type Culture Collection (Manassas, VA, USA) or the Japanese Collection of Research Bioresources Cell Bank (Osaka, Japan). Cells were cultured as described previously^35,36^. To obtain cell lysates, cultured cells (10^6^) were washed twice with phosphate-buffered saline and lysed with RIPA buffer (Thermo Fisher Scientific, Waltham, MA, USA). To obtain conditioned media, 10 ml of GC cells (10^5^/ml) was incubated in RPMI-1640 medium containing 10% fetal bovine serum in 10-cm dishes. Conditioned media were collected after 1, 2, or 3 days of incubation from different dishes for each day, centrifuged at 3000 rpm for 5 min, and stored at − 30 °C until analyzed^34^. Primary GC tissues were collected from 56 patients who underwent gastrectomy for GC without neoadjuvant chemotherapy and were processed as described previously^26^.

### ELISA

SDF4 concentrations in cell lysates, conditioned media, and sera were determined using a human SDF4 ELISA Kit (NBP2-75386, Novus Biologicals, Fontana, USA). We performed the ELISA assay of blood samples blinded to clinical information. Samples were tested in duplicate according to the manufacturer’s protocol, and the mean values are presented^37,38^.

### Immunohistochemistry

Immunohistochemical staining of SDF4 protein was performed as described previously^39–41^. In brief, sections were incubated for 1 h at room temperature with rabbit polyclonal anti-SDF4 antibody (Funakoshi, Tokyo, Japan) diluted 1:500 in antibody diluent (Dako, Carpinteria, CA, USA). The sections were then incubated with a biotinylated secondary antibody (SignalStain® Boost IHC Detection Reagent labeled with HRP; Cell Signaling Technology, Beverly, MA, USA) for 30 min. Antigen–antibody complexes were visualized by exposure to liquid 3,3′-diaminobenzidine (Nichirei, Tokyo, Japan) for 1 min. The method of evaluation for staining was shown in our previous study^41^.

### Statistical analysis

Group means of categorical and continuous variables were compared using χ2 and Mann–Whitney tests, respectively. Spearman’s rank correlation coefficient was used to assess the strength of correlations between two continuous variables. ROC curve analysis was performed to calculate AUC and determine the optimal cutoff values for distinguishing healthy controls from GC patients as well as for diagnostic performances of serum SDF4, CEA, and CA19-9 levels^42^. The Kaplan–Meier method with the log-rank test was employed to estimate OS, which was defined as the period between the day of curative gastrectomy and death^43^. OS calculations included all causes of death, and subjects lost to follow-up were censored. *P* < 0.05 was considered statistically significant. Statistical analysis was performed using JMP 16 software (SAS Institute, NC, USA).

### Supplementary Information

Supplementary Legends.

Supplementary Figure S1.

Supplementary Table S1.

## Data Availability

The data underlying this article will be shared on a reasonable request to the corresponding author.
