# Supplementary material for: Identification of stromal cell-derived factor 4 as a liquid biopsy-based diagnostic marker in solid cancers
Source: Sci Rep. 2023 Sep 20;13:15540. doi: 10.1038/s41598-023-42201-2 (PMC10511445; doi:10.1038/s41598-023-42201-2)
Supplement: Supplementary file 1 — Supplementary Legends. [file 41598_2023_42201_MOESM1_ESM.pdf]

Supplementary figure legend

Supplementary Fig. 1 Serum levels of SDF4 in healthy controls and patients with stage I, II, III, and IV GC stratified by sex (male and female).
