# Supplementary figures and images for: Identification of stromal cell-derived factor 4 as a liquid biopsy-based diagnostic marker in solid cancers
Source: Sci Rep. 2023 Sep 20;13:15540. doi: 10.1038/s41598-023-42201-2 (PMC10511445; doi:10.1038/s41598-023-42201-2)

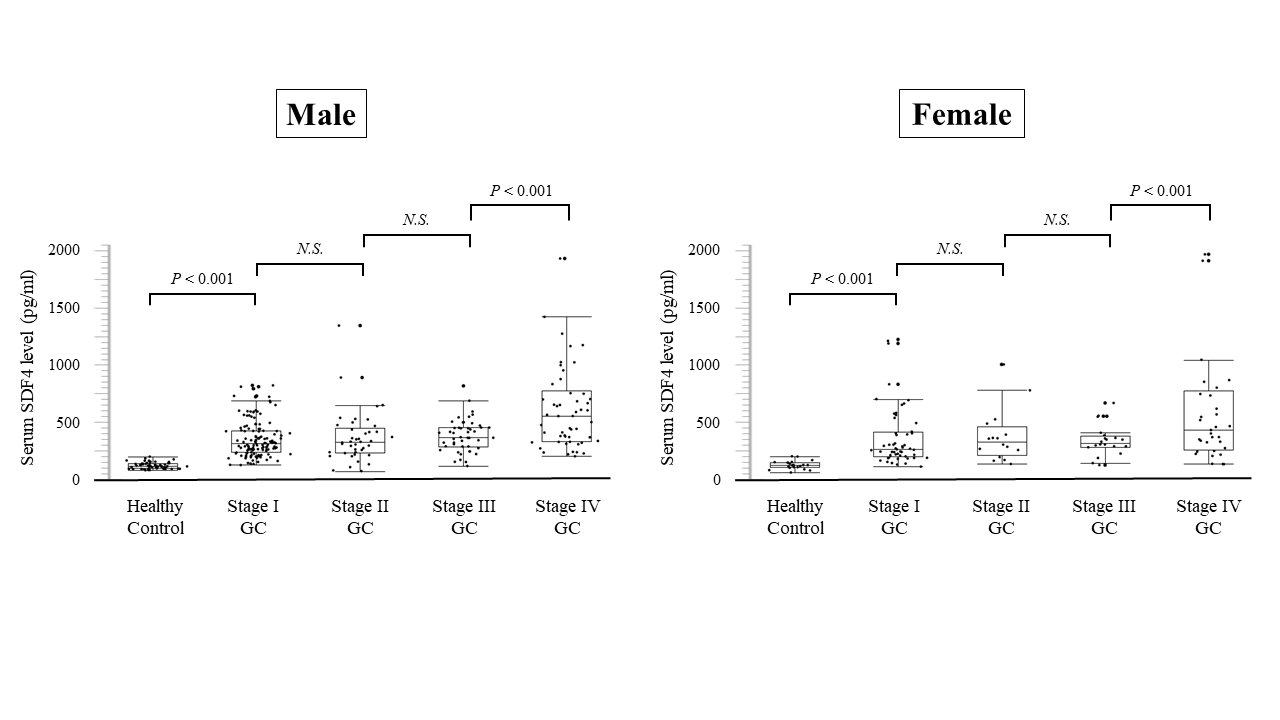

Supplement: Supplementary file 2 — Supplementary Figure S1. [file 41598_2023_42201_MOESM2_ESM.tif]
