# Supplementary material for: Identification of stromal cell-derived factor 4 as a liquid biopsy-based diagnostic marker in solid cancers
Source: Sci Rep. 2023 Sep 20;13:15540. doi: 10.1038/s41598-023-42201-2 (PMC10511445; doi:10.1038/s41598-023-42201-2)
Supplement: Supplementary file 3 — Supplementary Table S1. [file 41598_2023_42201_MOESM3_ESM.docx]

**Supplementary Table 1.** Diagnostic performance of 10 candidate proteins for detection of gastric cancer.

| Symbol | Full name | AUC | ELISA kit |
| --- | --- | --- | --- |
| SDF4 | stromal cell-derived factor 4 | 0.907 | NBP2-75386, Novus Biologicals, Fontana, USA |
| TINAGL1 | tubulointerstitial nephritis antigen like 1 | < 0.600 | EK1766, Boster Biological Technology, Pleasanton, USA |
| CTSV | cathepsin V | 0.836 | EK1929, Boster Biological Technology, Pleasanton, USA |
| DSC2 | desmocollin 2 | < 0.600 | ELH-DSC2-1, RayBiotech, Georgia, USA |
| LGMN | legumain | 0.601 | EK1566, Boster Biological Technology, Pleasanton, USA |
| PTPRK | protein tyrosine phosphatase receptor type K | 0.686 | F-EL-H0892, Elabscience, Texas, USA |
| AGRN | agrin | 0.846 | E3659Hu, BT LAB, Shanghai, China |
| CTSC | cathepsin C | < 0.600 | OKEH06920, Aviva Systems Biology, San Diego, USA |
| TFRC | transferrin receptor | < 0.600 | F-EL-H2345, Elabscience, Texas, USA |
| PSMA7 | proteasome 20S subunit alpha 7 | 0.682 | KTE61084, Abbkine, Georgia, USA |

*AUC* area under the curve, *ELISA* enzyme-linked immunosorbent assay.
